# Live Nativity and Brucellosis, Sicily

**DOI:** 10.3201/eid1212.060864

**Published:** 2006-12

**Authors:** Chiara Iaria, Filippo Ricciardi, Fernanda Marano, Giovanni Puglisi, Georgios Pappas, Antonio Cascio

**Affiliations:** * Dipartimento di Patologia Umana, Università di Messina, Messina, Italy;; † Dipartimento di Prevenzione Azienda Unità Sanitaria Locale 5, Messina, Italy;; ‡ Institute for Continuing Medical Education of Ioannina, Ioannina, Greece

**Keywords:** Brucellosis, Sicily, Brucella melitensis, letter

To the Editor: Worldwide, brucellosis remains a major zoonosis and an important cause of travel-associated illness (*1*). Brucellosis is transmitted to humans through the consumption of infected, unpasteurized, animal-milk products; direct contact with infected animal parts; or inhalation of infected aerosolized particles. We report an outbreak of brucellosis in a small village of the Ionic coast of Messina province (eastern Sicily).

In 2003, health authorities in the Messina province were notified of 29 cases of brucellosis; 18 of the patients were members of 9 different families. All patients had observed a Nativity pantomime that used live animals and was organized by the local population. Nativities in Sicily last ≈1 month, during which the sheep are milked, cheese and ricotta are produced, and these products are sold or offered fresh to tourists. All 29 patients had consumed dairy products: tuma cheese by 29 (100%) and tuma and ricotta by 16 (55%). No other risk factors for brucellosis were reported. Symptoms appeared after a median of 45 days (range 30–70). Eight patients were children (3 male), and 21 were adults (10 male). The median age of the children was 10.5 years (range 6–13) and of the adults, 42 years (range 16–67). Hospitalization was required for 5 patients. For 2 adults, brucellosis was complicated by spondylitis.

The real extent of the outbreak was likely large because in Sicily ≈60% of cases may go unreported. Furthermore, we report only the cases that occurred in the villages of Messina province and that were reported to health authorities; but tourists from many other areas in Italy and some from outside Italy generally attend such events. Southern Italy has commonly been implicated as a venue for travel-associated brucellosis (*2*).

In Italy, the overall incidence of brucellosis has gradually declined in the past 30 years, especially in northern Italy, where the disease is now reported only sporadically. This trend, however, has led to an increase in the percentage of total cases in Italians reported from the southern provinces of Calabria, Campagna, Puglia, and Sicily; of the 520 cases reported in 2003, 488 (93.8%) were reported from 4 southern regions, compared with 63.7% in 1994. Sicily alone reported 57.6% of the 2003 cases and for the past decade has had an average annual incidence of >100 cases per million (*1**,**3*). The disease is almost always caused by *Brucella melitensis* (*4*). The southern localization of the disease in Italy is obviously related to the relative high prevalence of infections in sheep and goats (*5*). Ovine and caprine population density is higher in the southern regions of Italy than in the rest of the country (*6*).

The Italian brucellosis eradication plan consists of a test-and-slaughter practice. However, in Sicily a vaccination campaign with *B. melitensis* Rev.1 strain has been started. In 2003, ≈99% of the stock farms were tested, and ≈18% of them were infected (*3*). The major problems reside with small flocks that undergo frequent transhumance (seasonal movement of herds between regions with different climates) in isolated regions where testing by veterinarians is difficult or avoided by the owners (a typical drawback of test-and-slaughter practices).

Technically, ricotta is not a cheese, but rather is a cheese by-product. The name "ricotta" means cooked again, referencing the production method. Ricotta is made from whey drained from tuma, provolone, and other cheeses. Heat is then used to separate, by precipitation, the remaining albumin from the whey left after making lactic acid/rennet–precipitated cheeses. It is eaten as is or used for food seasoning (e.g., classic Italian lasagna and ravioli). A cream made of sieved ricotta and sugar is used to prepare many desserts, like cannoli and cassata cake. Being cooked 2 times, ricotta should not contain viable *Brucella* organisms; however, shepherds sprinkle fresh milk on wicker baskets to refresh the ricotta they contain, thereby contaminating the product.

Tuma is a typical Sicilian fresh cheese made from sheep's milk. It has a cylindrical appearance and is sold fresh, no more than 2 days old. It has no crust, and the dough is white or ivory-white without holes. The texture is very soft, tender, and wet. It is generally served with ham, wines, and fruits as a table cheese.

Tuma cheese should be considered as the major vehicle of *B. melitensis* infection in Sicily. Although most similar dairy products produced in Sicily are derived from organized dairy companies and have been pasteurized, traditional delicacies from small villages may still cause brucellosis outbreaks.
